# Is the Thames Pernicious?
*Quarterly Report on the Sanitary Condition of St. Leonard's, Shoreditch, for the Quarter ending September 26th, 1857. By R. Barnes, M.D.


**Published:** 1858-04

**Authors:** 


### IS THE THAMES PERNICIOUS?*

Dr. Barnes makes the succeeding remarks on the influence of sewerage on the population:

"It is a momentous question, involving perhaps some millions in taxation—to be either wasted or saved—whether the influence of the summer heat upon the waters of the Thames be such as to empoison the atmosphere of London, and so to increase the mortality of the inhabitants? Never before have we enjoyed equal facilities for observing the influence of sewage and of high temperature on the Thames as a cause of disease. The temperature of the Thames has rarely been so high. The quantity of sewage was never so great. The population has vastly increased; and the excreta of many thousands which were formerly received into cesspools, have been, within the last eighteen months, added to the sewage flowing into the river. The eminent engineers appointed by Sir Benjamin Hall to report upon the main drainage of the metropolis, lay it down as an axiom, as the unquestioned justification for gigantic works, and commensurate expenditure, having for their object the 'dispollution of the Thames,'—

"'That the influence of the sewage upon the river is pernicious.'

"In what way the influence of the sewage is pernicious to the river it would be foreign to my duty to inquire. I presume it to be meant 'that the influence of the sewage poured into the river is pernicious to the health of the inhabitants of London.' I will not assert that this influence, if any, is not pernicious to health, but I do most emphatically deny that any definite proof that it is pernicious has been produced. The question and the consequences are too weighty to be decided by declamation or by prejudice. It must be decided by facts. Where are those facts? If the theory be true, we ought to trace the deadly influence of the river. First, and in the severest degree, amongst those who live on its waters.

* Quarterly Report on the Sanitary Condition of St. Leonard's, Shoreditch, for the Quarter ending September 26th, 1857. By R. Barnes, M.D.

Second, in those who dwell near its shores. Third, we ought to find comparative immunity from fever and diarrhœa amongst those who live at a distance from the river. These are points that admit of being determined by observation; by the comparison of the returns of the Registrar-General, and of the weekly register of new cases of sickness, compiled by the Association of Medical Officers of Health. Chemistry and microscopy may indeed prove the existence of a small proportion of living and dead organic matter in Thames water. This kind of proof is all that has been advanced in the elaborate mass of documents that form the appendix to the report of the referees on the main drainage of the metropolis. But to prove the actual presence of this organic matter, is a different thing from proving that it acts perniciously upon the health of the population. The effect of the Thames upon the public health is not to be decided by chemical or engineering science. Nor ought it to be taken for granted. It is a question for medical observation and statistical analysis. The evidence of these has not been called.

"From special opportunities of observing the forms and progress of disease prevalent on the Thames; from careful inquiry into the origin of those diseases; from a comparison of the sickness of the Thames with the sickness of Shoreditch; and from the periodical examination of the water of the Thames under the varying influences of tides, wind, rainfall, and temperature, in which I have been aided by Dr. Odling, the medical officer of health for Lambeth, the conclusion has been forced upon my mind that great exaggeration, if not a total misapprehension, prevails upon the subject of the pernicious influence of the Thames upon the public health.

"I call attention to one fact: since the replacement of the old *Dreadnought* by the present ship, now nine months ago, not a case of fever has originated on board this floating hospital."

### MISCELLANEOUS NOTICES.

From the remaining books and tracts in hand, our space will allow us to notice only a few, and these briefly. Dr. Mackay, in a pamphlet entitled *Notes on the Cholera at Varna in* 1854, gives some striking illustrations as to the contagious character of the great Asiatic malady. Mr. Nourse, in a paper entitled *A Short and Plain History of Cholera, its Causes, and Prevention; written for popular use,* gives in a simple and connected form a very useful history of the disease named. His work may be read by the public at large with advantage. Dr. Murphy has a little work, entitled *What is Puerperal Fever?* We regret that the learned professor propounds the question without attempting to answer it. Dr. Lankester, as Medical Officer of St. James's, Westminster, has issued in a tract a series of *Plain Directions for the Preservation of Health, and the Prevention of the Spread of Contagious Diseases.* Tractarianism is here turned to some real service; for the rules are legible, and the poorest may read.

